# Quantifying Ant Activity Using Vibration Measurements

**DOI:** 10.1371/journal.pone.0090902

**Published:** 2014-03-21

**Authors:** Sebastian Oberst, Enrique Nava Baro, Joseph C. S. Lai, Theodore A. Evans

**Affiliations:** 1 Acoustics & Vibration Unit, School of Engineering and Information Technology, University of New South Wales Canberra, Canberra, Australian Capital Territory, Australia; 2 Departamento de Ingeniera de Comunicaciones, Escuela Tecnica Supérior de Ingeniería Telecomunicación, Campus de Teatinos s/n, Universidad de Málaga, Málaga, Spain; 3 Department of Biological Sciences, National University of Singapore, Singapore, Singapore; University of Sussex, United Kingdom

## Abstract

Ant behaviour is of great interest due to their sociality. Ant behaviour is typically observed visually, however there are many circumstances where visual observation is not possible. It may be possible to assess ant behaviour using vibration signals produced by their physical movement. We demonstrate through a series of bioassays with different stimuli that the level of activity of meat ants (*Iridomyrmex purpureus*) can be quantified using vibrations, corresponding to observations with video. We found that ants exposed to physical shaking produced the highest average vibration amplitudes followed by ants with stones to drag, then ants with neighbours, illuminated ants and ants in darkness. In addition, we devised a novel method based on wavelet decomposition to separate the vibration signal owing to the initial ant behaviour from the substrate response, which will allow signals recorded from different substrates to be compared directly. Our results indicate the potential to use vibration signals to classify some ant behaviours in situations where visual observation could be difficult.

## Introduction

Ant behaviour is of considerable interest, due to their sociality. A large body of information now exists on life cycles, reproduction, foraging, construction, ecology and communication [1]–[6]. All ant behaviour is observed visually, either directly by human eye or using cameras. Some electronic observation is automated with image processing techniques [7]. However, ants can be difficult to observe at various times and situations. For example, most ants build nests in soil, under stones, in wood, under bark and similar situations, which are difficult to access; some ants are nocturnal [3],[4],[6]. Therefore alternative observation techniques may be useful.

All behaviour that involves physical movement will generate vibrations or acoustic signals. These signals are transmitted through the air (sound) or travel through a solid substrate (vibrations) and may be used to detect movement. This could be especially useful for ants in difficult to observe situations. Moreover, ants are known to generate and detect vibration signals [8]–[14], suggesting that further exploration of vibration signals may prove useful to behavioural research in ants. In addition, there has been no work to extract the excitation signals produced by ants from their physical movement, from the response signals produced by the substrates where the signals are recorded.

Here we test the possibility of using vibration signals as a tool to assess ant activity. We placed ants in containers in the laboratory and exposed them to different situations in order to trigger different types of physical movements. We observed ant behaviours and recorded the vibrations generated for each situation, then performed a detailed analysis of the vibration signals to characterise them, and determine whether the differences between signals would be large enough to identify the physical movements. Also, we developed a novel method to extract the excitation signals from the substrate's response using wavelet filtering and signal de-convolution in an adaptive linear mechanical model. This will allow comparison of recorded signals from different substrates.

## Materials and Methods

### Study species and general apparatus

We used the common and widely distributed (not endangered) Australian species *Iridomyrmex purpureus* (subfamily Dolichoderinae). It was chosen because it is common, relatively large (7 to 9 mm long), hence easy to handle and observe, and ecologically and economically important [15]–[18]. We used ants from three different colonies each at least 3 km apart from CSIRO at Black Mountain and at UNSW Canberra near Mt. Pleasant. We collected ants from the field as required; used them in experiments for 2–6 days, feeding them honey water, then we returned them to their colonies. A specific permission to collect specimen was not required at either locations as the number of collected insects was very small, their nests were not damaged and insects were not killed but returned after the experiments whenever possible.

**Figure 1:**
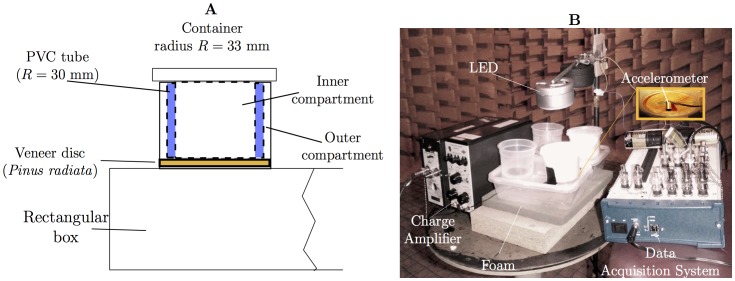
The experimental setup. **A**, Schematic of ant-box setup with PVC tube, veneer disc, lid and container with inner compartment and outer ring segment to house insects; and **B**, photo of the experimental setup in an anechoic chamber [22].

We built the apparatus used in all experiments from food-grade polypropylene boxes as they were cheap, plentiful and thus easy to replace between experiments. The basic unit was the ‘ant-box’ (schematic in **Figure 1**), which consisted of a rectangular plastic container (155 mm×105 mm×40 mm), with cylindrical containers (polypropylene, radius 33 mm, height 41 mm) surmounted and glued on the lid of the rectangular container. We cut circular holes (radii 25 mm) through the base of the cylindrical containers and the lid of the rectangular container (radii 30 mm). A wooden veneer disc (*Pinus radiata*, radius 30 mm, thickness 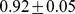 mm) was placed over each circular hole. We wanted to avoid using glue on the wood, yet keep the wood firmly against the polypropylene box without any gaps. Therefore we placed a PVC tube (radius 30 mm, height 41 mm) inside the cylindrical container to secure the wood in place by pressure from the lid of the cylindrical container. This created two spaces inside the cylindrical container: an ‘inner’ compartment inside the PVC tube, and an ‘outer compartment’ between the outside of the PVC tube and the cylindrical container. The inside surface of the cylindrical container and the outside surface of the PVC tube were sanded to give the ants grip to walk on. The vibration response of the veneer disc in the ant-box was analysed separately (Figure S1) with peak frequencies found at around 455 and 495 Hz (Figure S2).

We used 15 test ants for the vibration bioassays, and we used new ants for each replicate. We placed the ants in the inner compartment of the cylindrical container (Figure 1**A**). We positioned ant-boxes on foam to isolate environmental vibrations (Figure 1**B**). We recorded ant activity and its vibrations in an anechoic chamber (3.5 m×3.5 m×3.5 m) with a lower cut-off frequency of 150 Hz and an average background sound level of less than 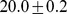 dB. We maintained room temperature at 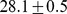
°C with an electric oil heater (Sunair NHS9); we did not attempt to control relative humidity, which was 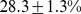.

### Vibration bioassays

The purpose of the vibration bioassays was to ascertain whether the level of ant behaviour could be quantified by their vibration responses to various stimuli. We used miniature accelerometers to measure the veneer disc's response to ant excitations (Figure 1**B**). We attached an accelerometer (B&K model 4374, sensitivity 0.150 and 0.158 pC/ms) with bee's wax to the centre of each veneer disc (one served as the control and the other served as the treatment for each ant-box), and acquired the signal using a charge amplifier (B&K model 2635) with a National Instruments data acquisition system (NI USB 6251) and LabView Signal Express, Sound and Vibration Assistant 2.5.0 on a Toshiba Tecra notebook. We calibrated the accelerometers at 1 kHz with an average background noise floor power spectral density of 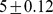 dB/Hz re 0.1 mV. Each power spectral density measurement consisted of an average of 2500 power spectral densities which were taken every second to estimate the mean power using a sampling frequency of 7 kHz [19] and a linear phase FIR (finite impulse response) filter (up to 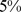 of the normalised Nynquist frequency) with Kaiser window (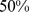 overlap).

We observed the following ant behaviours: walking; carrying stones; falling from the PVC tube; scratching or biting the PVC tube; agitated shaking along their longitudinal axis, tapping of hind legs on the veneer, self-grooming, and interactions between ants such as antennation and trophallaxis. We used these observations to design the following five bioassay treatments:

- ‘Illuminated’ ( = control). We applied no other external excitation to the ant-boxes other than illumination (n = 62 replicates);
- ‘Shaken’. We tapped and shook the ant-box gently up and down (about 150 mm) for 10 s without causing any obvious damage to the ants; illuminated (12 replicates);
- ‘Stones’. We collected some of the small stones that cover meat ant nests [20] and placed 12 stones (average weight 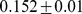 g, 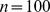) to the inner compartment of the cylindrical container; illuminated (15 replicates);
- ‘Neighbours’. We placed three ants (nestmates of the test ants) in the outer compartment of the cylindrical container to provide an ant-neighbour signal; illuminated (23 replicates);
- ‘Darkness’. We applied no external excitation, and no illumination (same as control but without light; 15 replicates). We assumed this treatment would have the lowest activity because meat ants are diurnal.

In order to calm the ants after handling, we started recording two minutes after setting up the containers and applying the excitation. We illuminated ant-boxes for all treatments (except ‘Darkness’) with 25 cold-white LEDs, connected in parallel to a battery pack, which we used to avoid the 50 Hz flickering from the AC power. This gave an average light level of 3775 intensity counts at 460 nm.

We obtained the distribution over the number of recorded signals at each frequency, and we tested each for normality using a Lilliefors goodness of fit test at an alpha of 0.01. We computed the average power spectral density (PSD) from the geometric mean data as it weighted different treatments equally. We conducted the tests on different days with various colonies and different groups of ants from each colony, but always with the same experimental setup and ant species, and we assumed the data obtained would have the IID (independent, identically distributed) properties [21].

We used unbalanced ANOVAs to compare the recorded signals from the treatments. First we tested the distributions of the recorded control signals (‘Illuminated’) for differences between experiments; second we tested the mean peak power spectral density for significant differences in power per frequency between control and treatment signals.

### Analysis of ant vibration signals

The purpose of the analysis was to separate the excitation signal caused by the ant behaviour from the substrate (veneer disc) response in time series data, and thereby determine whether the excitation signal could be used to classify ant behaviour during bioassays. We filmed the ants with a digital camera (Olympus Tough with 12 MP) so that we could correlate ant behaviour with the recorded vibration signal.

**Figure 2:**
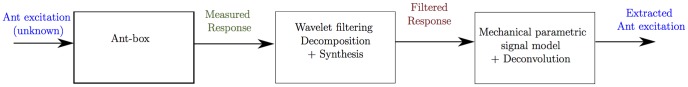
Excitation signal extraction. Schematic process of excitation signal extraction based on the experimental system response (thick lined box).

We used a two step procedure for each ant behaviour to extract the ant excitation signal from the recorded substrate response (Figure 2). First, we used the measured vibration response at the centre of the veneer disc (measured response) and then via wavelet decomposition, we synthesised the dominant vibration waveforms to a ‘wavelet-filtered’ response. Second, we used a linear parametric model of this ‘wavelet-filtered’ response and extracted the excitation signal caused by the ants' activity.

We extracted the substrate response signal's waveform using wavelet toolbox in Matlab R2011a; in particular we used the Continuous Wavelet Transform (CWT) for qualitative assessment of the signal and the Discrete Wavelet Transform (DWT) for quantitative decomposition of the substrate response due to ant excitation (details in section B in File S1 and [22]). We applied a scalogram as visual assurance of proper selection of scales based on the CWT, using up to 128 scales and a Morlet mother wavelet [23], [24]. We used this qualitative information to decompose the substrate response with DWT (Meyer mother wavelet, 6-level dyadic decomposition), and re-assembled them with only the most important scales (which had 95% of the signal's energy). Thus, noise and distortion were reduced while information of the original substrate response signal was preserved (c.f. [22]).

We used a linear model of the veneer disc with one or two exponentially damped sinusoids in noise and the matrix pencil method [25] to estimate the model parameters (section C in File S1 and [22]). We assumed that a two damped sinusoid model (5th order) would fit the experimental data better when more than one vibration mode of the wood was excited. We also assumed that the excitation could be described by an impact model; except for scratching or biting, when we used two sub-models for which the amplitude first grew and then decayed second (oscillating increasing-decreasing terms) with an envelope similar to that of frictional sounds [26].

## Results

### Vibration bioassays

**Figure 3:**
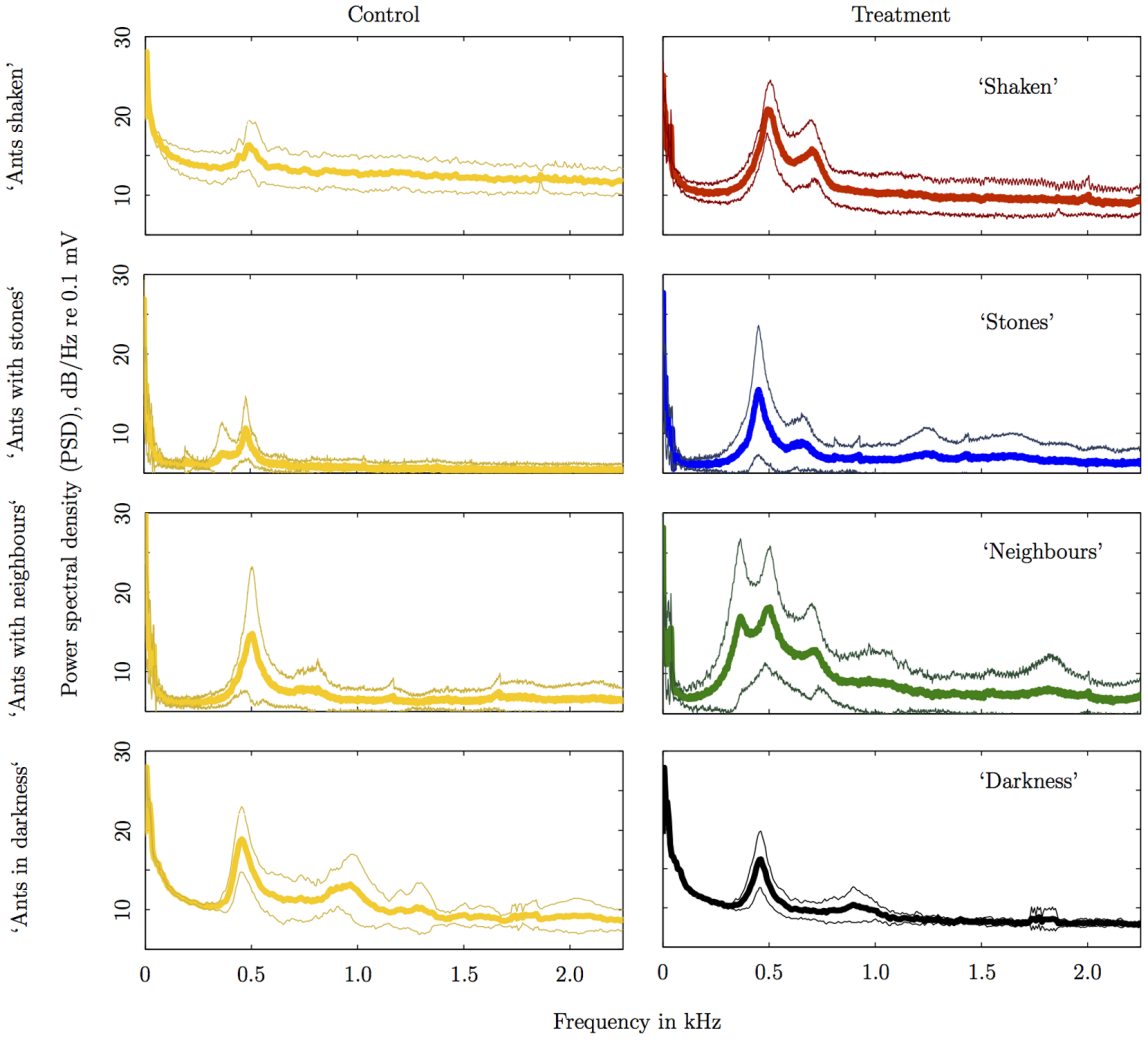
Analysis of average power spectral density. Thin lines are one standard deviation away from the average (thick line); treatments: ‘Shaken’, (vs. illuminated as control) (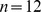); ‘Stones’ (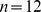); ‘Neighbours’ (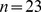); and ‘Darkness’ (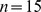).

The power spectral density (PSD) of each recorded acceleration signal from the veneer disc was normally distributed for frequencies less than 2.2 kHz (Lilliefors goodness of fit test, 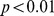). The average power spectral density with its standard deviation for frequencies up to 2.2 kHz is shown in Figure 3. In all treatments, the PSD decreased as frequency increased with some frequency peaks corresponding to the resonance modes of the veneer disc (first mode at around 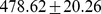 Hz; 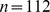, Figure S2) excited by ant behaviour. Comparison of the controls with treatments in the unbalanced ANOVA found that the mean over all four distributions were not significantly different (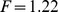, 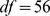, 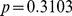).

**Figure 4:**
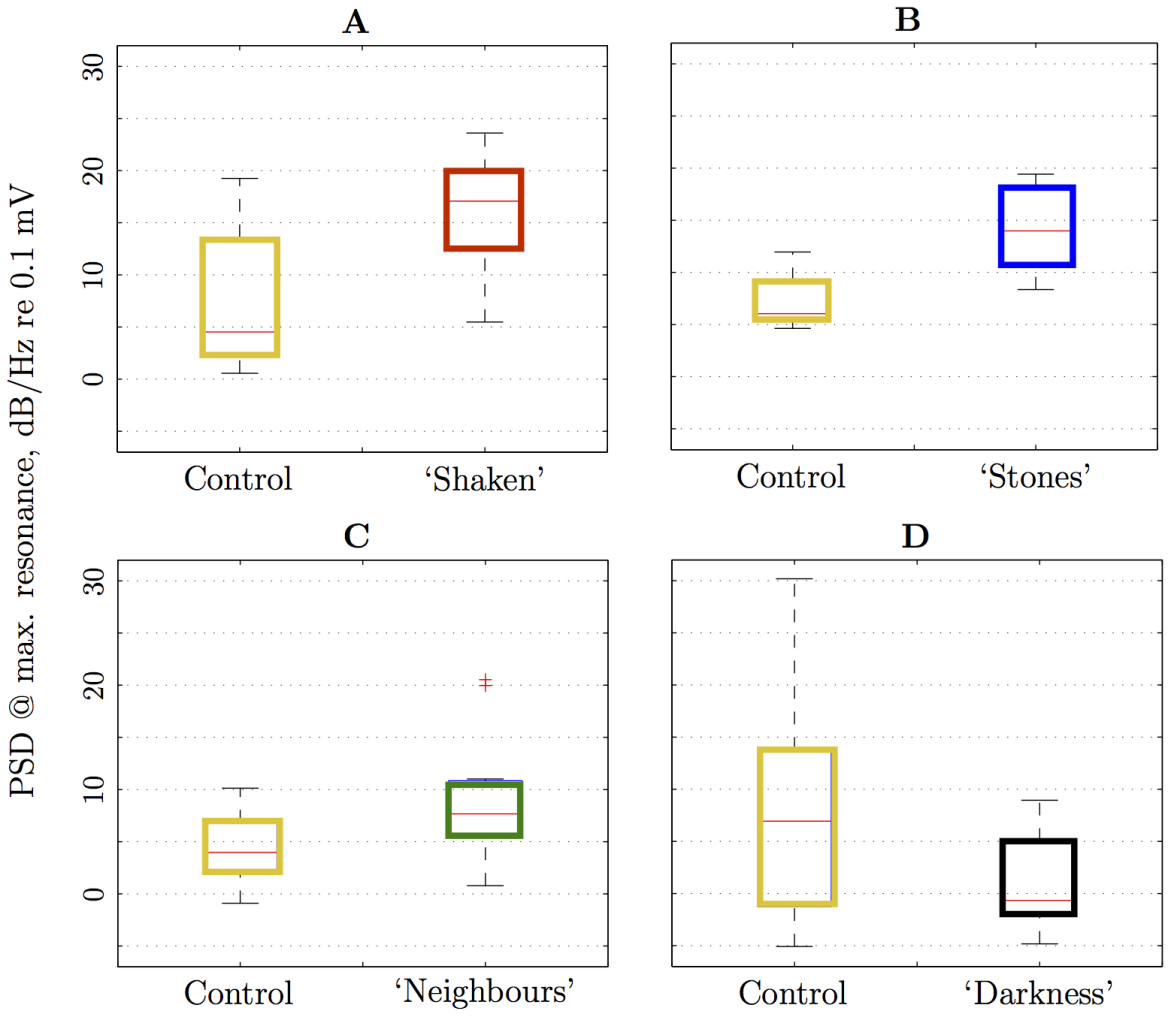
Box-plots of peak power spectral density (acceleration). Measurement for various treatments against control (‘Illuminated’): **A** ‘Shaken’; **B** ‘Stones’; **C** ‘Neighbours’; and **D** ‘Darkness’. Each box gives the lower quartile, median, and upper quartile values; whiskers correspond to the most extreme data values within 1.5 times the inter-quartile range are depicted from the ends of the box; outliers are data with values beyond the ends of the whiskers and displayed with a red+sign [29].

The average vibration acceleration PSD at the dominant frequency (corresponding to the maximum amplitude for frequencies greater than 100 Hz; determined from Figure 3) is shown in Figure 4. The highest activity recorded was in treatment ‘Shaken’, followed by ‘Stones’, then by ‘Neighbours’, ‘Illuminated’ and ‘Darkness’ had the lowest activity (Figure 4). The PSD of the treatments were always significantly different from their corresponding controls: ‘Shaken’ (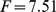, 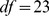, 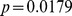), ‘Stones (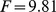, 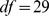, 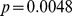), ‘Neighbours’ (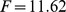, 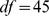, 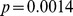) and ‘Darkness’ (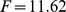, 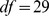, 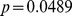).

### Analysis of ant vibration signals

**Figure 5:**
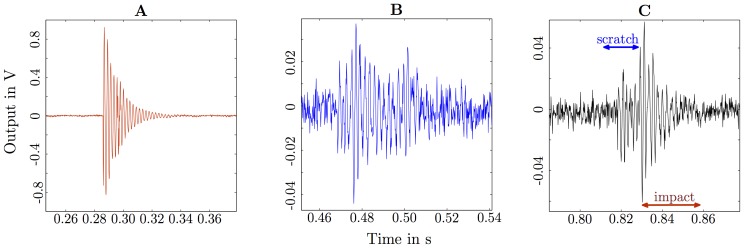
Typical vibration signals measured at the veneer disc. **A**, impact due to ants falling onto the veneer disc; **B**, scratching/biting responses; and **C**, initial scratching of a stone carried then dropped resulting in a subsequent impact; the strongest signal-to-noise ratio has been obtained for the impact **A** the weakest for signal **C** [22].

There were two types of signals or signal combinations detected. The first signal type was an impact excitation, observed when ants fell off the PVC tube onto the veneer disc (Figure 5**A**) or when stones were dropped by ants. The second signal type was due to scratching or biting, either (and mainly) when ants bit on the edge of the PVC tube (Figure 5**B**), or when ants dragged stones over the veneer disc. A combined signal type occurred when a stone was dragged then dropped (Figure 5**C**). Other signals (e.g. walking) were rarely confirmed as they were masked by the background noise.

**Figure 6:**
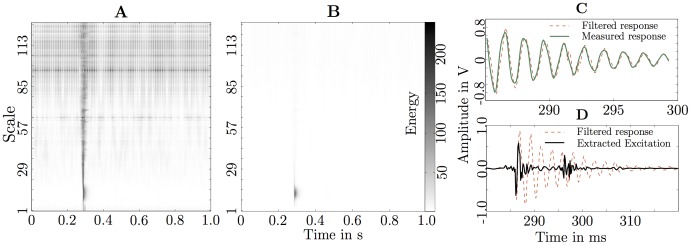
Analysis of impact signal (Figure 5A). **A**, Continuous wavelet analysed data normalised with respect to scale energy; **B**, Continuous wavelet analysed data non-normalised; **C**, Measured response and discrete wavelet filtered synthesised response; and **D**, filtered response vs impact as extracted excitation using a deconvolution filter.

The scalogram (with energy normalised by the maximum for each scale) in Figure 6**A** showed that the energy is non-stationary and is distributed over all 127 scales (starting from at about 0.3 s) for the recorded vibration signal in Figure 5**A**. The non-normalised energy values in Figure 6**B** showed that most of the energy is contained in the first 19 scales. The ant excitation signal input, extracted after applying a 5th order model (from the first 19 scales, filtered response, Figure 2) and deconvolution to the measured signal at the veneer disc, resembled two impact signals at 0.2853 s and 0.2976 s (Figure 5**D**). The most likely explanation for two impacts was a stone bouncing on the veneer disc.

The observed behaviours dominant in each treatment are listed in Table 1. The three behaviours that were dominant (falling, scratching/biting, dragging stones) were measured reliably, whereas walking was measured only sometimes. Behaviours that created weak signals, that is less than the noise floor (vibrating, tapping hind legs, grooming, antennation and trophallaxis) could not be measured. The extraction of excitation signals belonging to the scratching/biting response (Figure 5**B**) and the stone carrying/dropping response (Figure 5**C**) are presented in Figure S3.

**Table 1:** Observed behaviours in experimental setup.

Observed behaviour	Signal measurable	Dominated in
Walking	***✗***	‘Illuminated’
Tapping hind legs	***✗***	‘Illuminated’
Grooming	***✗***	‘Illuminated’
Antennation/feeding	***✗***	‘Illuminated’
Vibrating	***✗***	‘Stones’
Falling	**✓**	‘Shaken’
Scratching/biting	**✓**	‘Neighbours’
Dragging stone	**✓**	‘Stones’

Behaviours which have a ***×*** could not be assigned to a waveform; dominance in treatments, for the treatment in darkness nothing has been observed.

## Discussion

We tested the possibility of using vibration signals created by ant activities to record ant behaviour. We used a novel systematic approach that subjected the ants to various laboratory conditions. Although we used an artificial set up, some of the observed behaviours were common and natural, such as walking, climbing, grooming, antennation and trophallaxis. We considered dragging the stones to be natural as well, as the mounds of *I. purpureus* are covered by stones, and must be moved there by the ants. Of course, some of the ant behaviours were not natural and due to the experimental setup (falling after the ant-box was shaken, scratching/biting the PVC tube), nevertheless these behaviours or equivalents may occur under natural conditions.

We observed two new behaviours during our study. The first was a vibration signal immediately after illumination was applied: the vibration signal was created by oscillating the body along the ant's longitudinal axis. This usually occurred when facing a nest mate and may be an alarm signal. This vibrational signal has not been reported for *I. purpureus* in the literature, however vibrational alarm signals have been observed for other species of ants [27]. The second signal was a tapping motion with the hind leg on the ground or vibrating in the air; again this signal has not been reported for *I. purpureus* and it is not clear if this motion is related to stridulatory motion as observed in other ants [3] or if it is some kind of displacement activity (behaviour in captivity) [28].

Our treatment types did affect ant behaviour, primarily the intensity of activity. We observed that ants escalated and reduced their activity quickly in all treatment types. Perhaps unsurprisingly, the highest activity recorded was from the ‘Shaken’ treatment ants, in which the ants walked vigorously, climbed the PVC tube and dropped onto the veneer disc. The second highest activity was recorded from ‘Stones’, perhaps provoking nest maintenance. The addition of neighbour ants initiated a positive reaction measured by higher ant activity, perhaps as the signals from the neighbour ants enticed the test ants to find their nestmates, which resulted in biting and scraping noises mainly from the tube wall with no obvious feedback loops being identified in the experiment. Finally, the lowest activity was recorded from ants in the ‘Darkness’ treatment, again, unsurprisingly for a diurnal species.

We were able to distinguish three activity types reliably using the wavelet analysis (falling, scratching/biting, dragging stones), with a fourth (walking) less so. We were not able to distinguish various other observed behaviours (vibrating, tapping hind legs, grooming, antennation and trophallaxis), probably because the signals they created were weak, and thus hidden by background noise. The lower scales (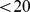) of the three distinguishable signals contained most of the energy. Our preliminary results show that the signal processing method is capable of classifying signals such as falling ants. However, for other ant behaviours such as scratching, a different mechanical model for the specific behaviour had to be developed.

Some of the behaviours we were not able to distinguish are of biological interest. Therefore it would be useful to continue this approach, but using more sensitive equipment. The signals arising from vibrating, tapping hind legs, grooming, antennation etc. may be isolated by using a more sensitive measuring device such as a laser vibrometer in conjunction with the appropriate absorption of disturbing environmental vibrations. This would increase the potential and utility of vibration measurements in bioassays aimed at studying ant behaviour.

## Supporting Information

Figure S1 **Experimental setup to measure the mobility of the veneer disc in the ant-box's lid.** Scanning laser vibrometer (PSV-400); loudspeaker; and ant-box minus rectangular container, Figure 1, main document.
(TIFF)

Figure S2
**Characterisation of veneer discs over mobilities.** Mobilities measured for systems **1**, **2** and **3**; 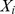 and 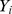 stand for the veneer discs' later use as control and treatment sides of system 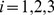, respectively.(TIFF)

Figure S3 **Analysis of signals.** Synthesised response of the model (filtered response, Figure 2 main document) and its de-convoluted signal (extracted excitation) for **A** the scratching sound only (Figure 5**B**, main document) and **B** the carrying and dropping of a stone (Figure 5**C**, main document).
(TIFF)

File S1 **Supporting Information.**
(PDF)
