# Supplementary figures and images for: Quantifying Ant Activity Using Vibration Measurements
Source: PLoS One. 2014 Mar 21;9(3):e90902. doi: 10.1371/journal.pone.0090902 (PMC3962336; doi:10.1371/journal.pone.0090902)

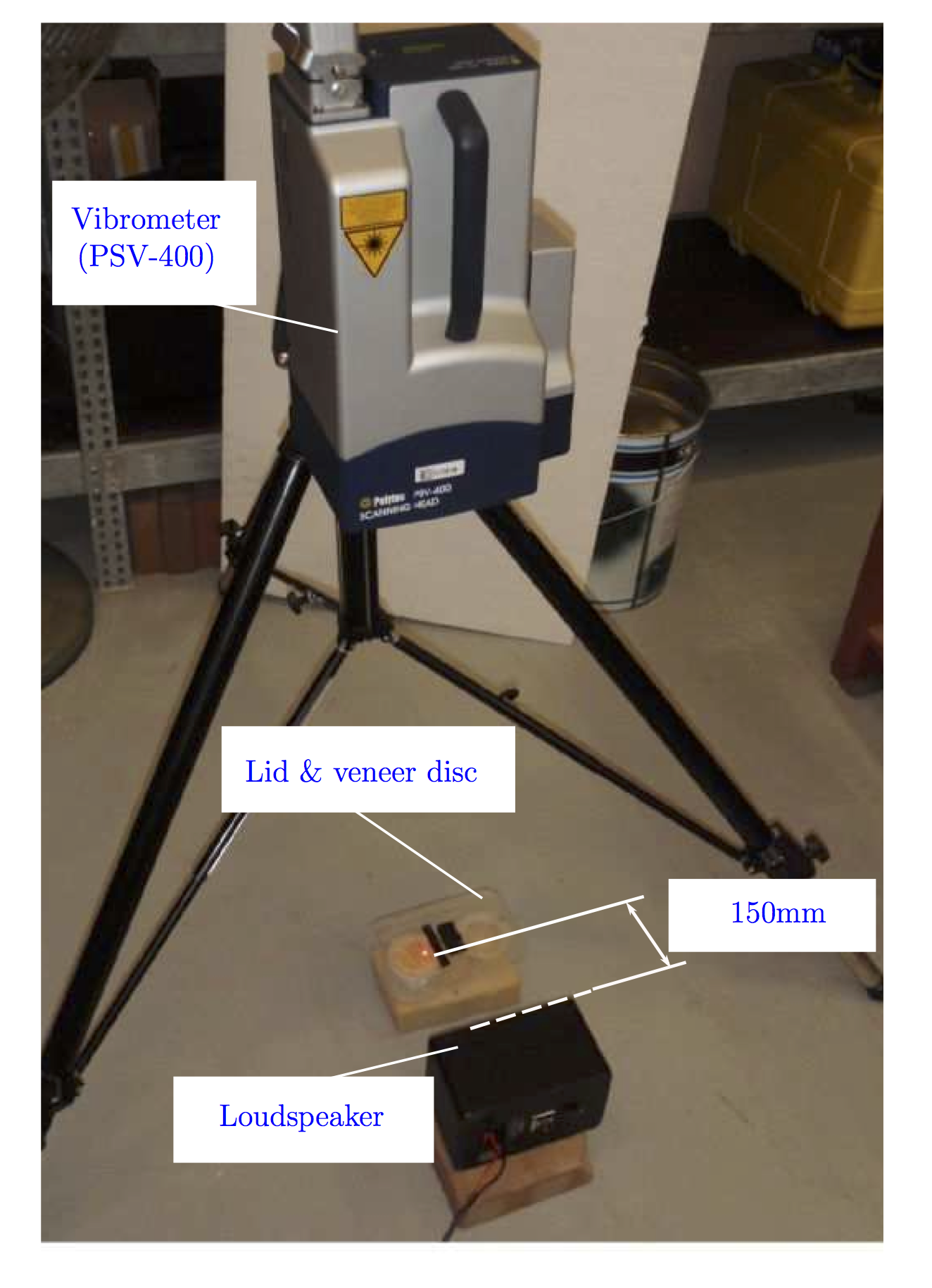

Supplement: Figure S1 — Experimental setup to measure the mobility of the veneer disc in the ant-box's lid. Scanning laser vibrometer (PSV-400); loudspeaker; and ant-box minus rectangular container, Figure 1, main document. (TIFF) [file pone.0090902.s001.tiff]

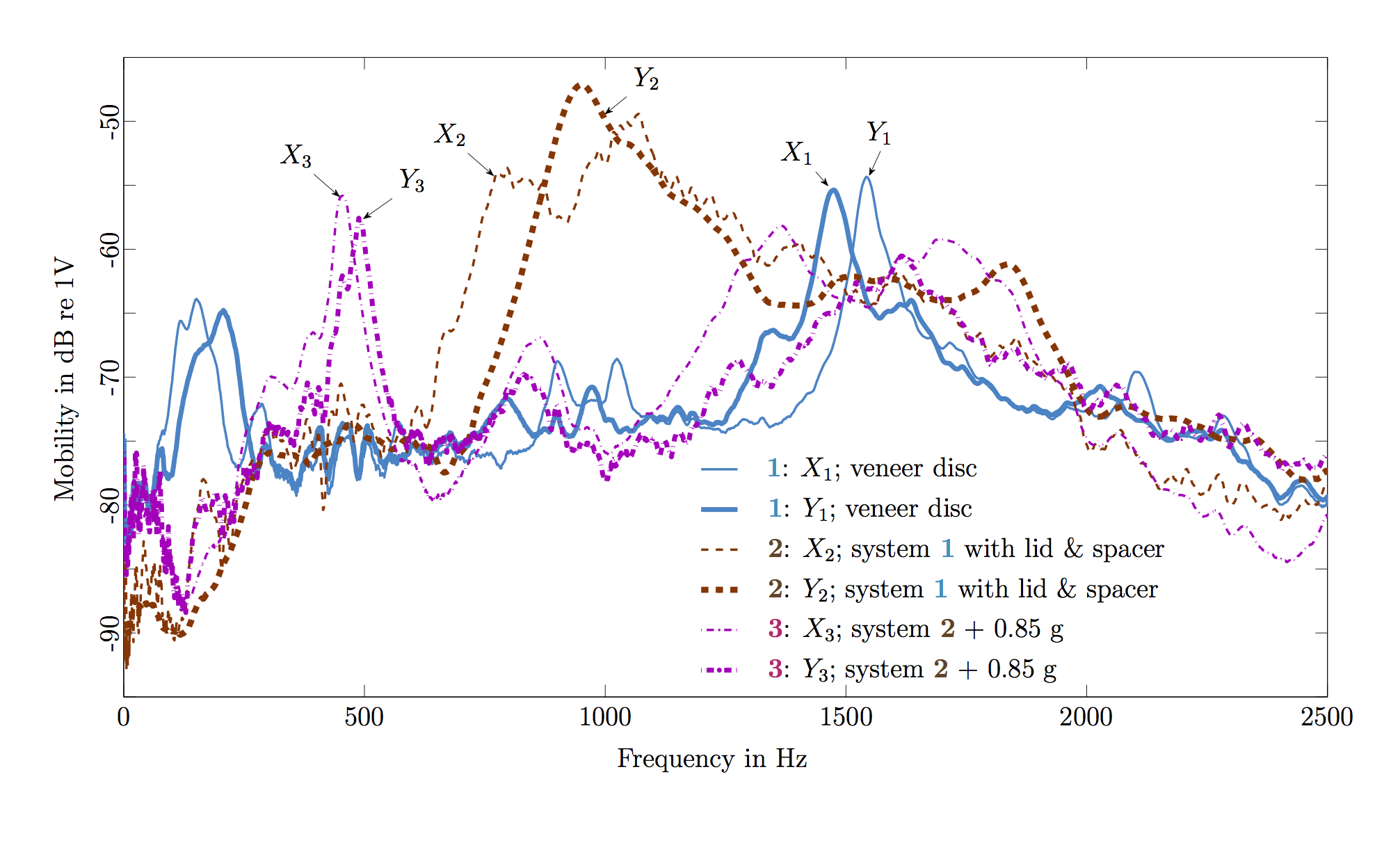

Supplement: Figure S2 — Characterisation of veneer discs over mobilities. Mobilities measured for systems 1, 2 and 3; and stand for the veneer discs' later use as control and treatment sides of system , respectively. (TIFF) [file pone.0090902.s002.tiff]

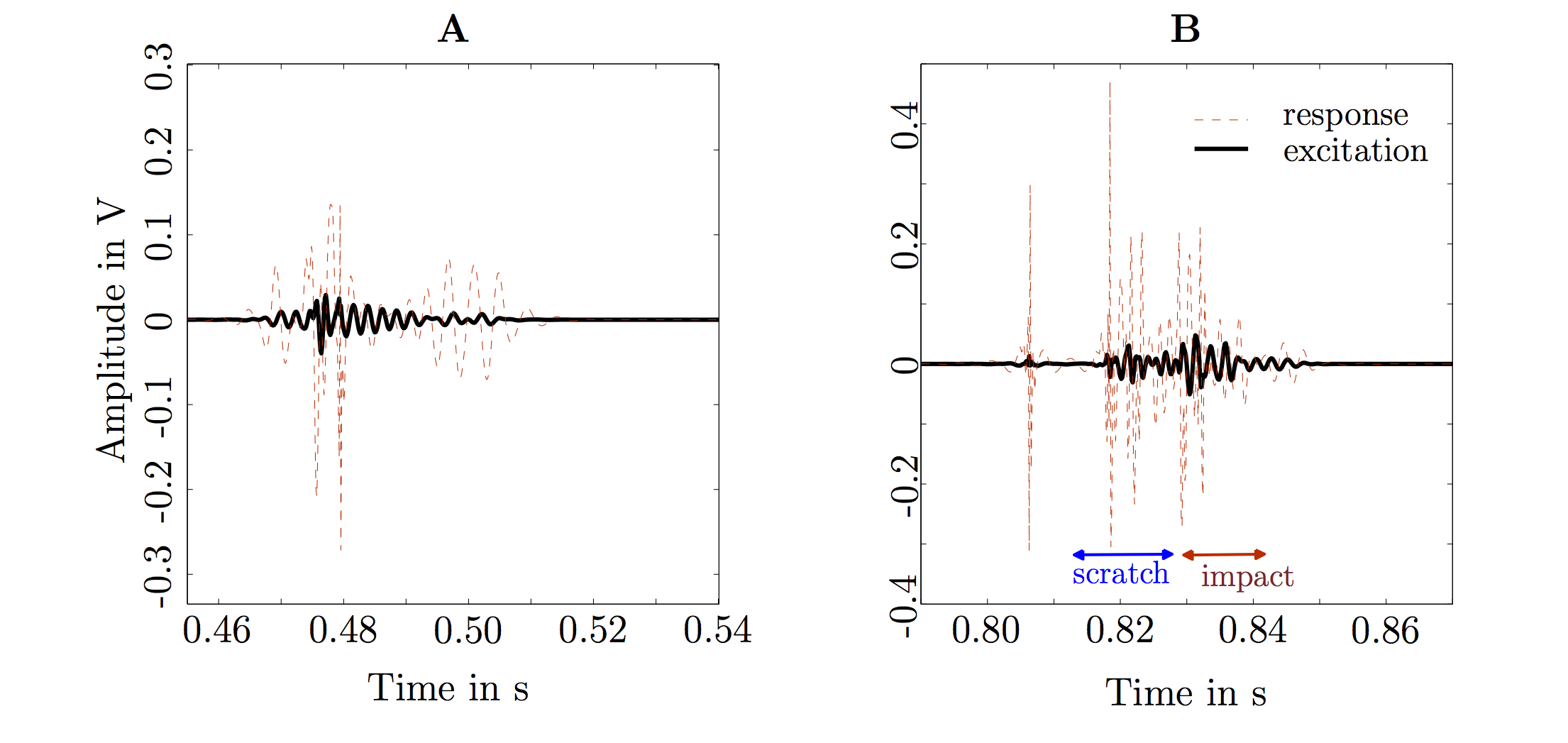

Supplement: Figure S3 — Analysis of signals. Synthesised response of the model (filtered response, Figure 2 main document) and its de-convoluted signal (extracted excitation) for A the scratching sound only (Figure 5B , main document) and B the carrying and dropping of a stone (Figure 5C , main document). (TIFF) [file pone.0090902.s003.tiff]
