# Supplementary material for: Quantifying Ant Activity Using Vibration Measurements
Source: PLoS One. 2014 Mar 21;9(3):e90902. doi: 10.1371/journal.pone.0090902 (PMC3962336; doi:10.1371/journal.pone.0090902)
Supplement: File S1 — Supporting Information. (PDF) [file pone.0090902.s004.pdf]

Supplementary Information A Preparatory experiment: Mobility of veneer disc

Materials and Methods

In order to use two assembled ant-boxes with different veneer discs in the vibration bioassay experiments the veneer discs' modal behaviour without ants has been assessed. The veneer discs had a weight difference of less than 4 %. The veneer disc in the ant-box **1** (used later on treatment side in container 1, called **X** in the following), had a weight of 1.4212 g; cf. veneer disc in the ant-box **2** (used later on control side in container 1, called **Y** in the following) of 1.3659 g. The second containers remained empty throughout the experiment.

Figure S1. Experimental setup to measure the mobility of the veneer disc in the ant-box's lid. Scanning laser vibrometer (PSV-400); loudspeaker; and ant-box minus rectangular container, Figure 1, main document.

The vibration response of the the veneer disc in the two ant-boxes were measured for three different systems, **1** veneer disc only, **2** veneer disc plus PVC tubes in ant-boxes and lid; and **3** as for **2** with an additional weight (Blue Tack, 0.849 g) in the middle of the veneer to mimic a load of both 15 ants (around 0.342 g,) plus the accelerometer load (mean of 0.7 g, $n = 5$). The setup as used for (3) but with accelerometers instead of Blue Tack would be the one used later on for the vibration bioassays with live ants.

Figure S2. Characterisation of veneer discs over mobilities. Mobilities measured for systems **1**, **2** and **3**; X_i and Y_i stand for the veneer discs' later use as control and treatment sides of system $i = 1, 2, 3$, respectively

The vibration velocities on the veneer discs' surfaces were measured with a laser vibrometer (Polytec PSV-400 & Polytec Analysis Software 8.8; Figure S1) using complex averaging ($n = 20$), a Hann window, anti-aliasing filter and a concentrical measurement grid of 81 nodes on each veneer disc. The empty ant-boxes were excited using a loudspeaker (Radioshack Realistic Minimus 7) via sweeping a signal of 0.3 s length ranging from 1 to 7000 Hz driven with 10 V delayed by 1/10 s. The loudspeaker was placed 150 mm away from the setup with the woofer centered on container 1 (n.b. the woofer's frequency response is given with $55 - 5000$ Hz). Non-contact excitation (loudspeaker) and measurement

(vibrometer) was preferred owing to the ant-boxes' light-weight structured lid-assemblies (about 157.71 g). As the distance between the speaker and the lid-assembly is 150 mm, the sound wave propagation is that of a spherical wave. Thus, as a first order approximation the lid-assembly was only excited by a plane wave for frequencies above 400 Hz [1]. The mobility was calculated as the ratio of the averaged measured vibration velocity relative to the voltage driving the loudspeaker (reference signal).

Results

Figure S2 gives the mobilities of systems **1**, **2** and **3**; X_i and Y_i stand for the veneer disc's later use as control and treatment side respectively for systems $i = \mathbf{1}, \mathbf{2}, \mathbf{3}$. The differences in the measured mobility between the two setups are very similar: 4.35% for system **1**, 10.35% for system **2**, and 6.75% for system **3**. The dominant mode of the veneer disc is the $(1, 0) = (m, n)$ mode, for system **3** 455.3 and 495.3 Hz, where m and n are the number of nodal circles and the number of radial nodal lines respectively [2]. It is expected that the ants will also excite this fundamental mode of the lid assembly (ant-box minus rectangular container, Figure 1 main document). While for system **2** the response is higher when the veneer disc is constrained to the lid assembly acting similar to a drum, the magnitude of the measured response for system **3** is reduced.

We acquired the laser vibrometer after we had finished the experiment with the B&K accelerometers (model 4374), partly because we realised the miniature accelerometers were not sensitive enough for some of the ant behaviours. However, in order to conduct the bioassays two laser vibrometers would be required and cooling of two laser heads (due to elevated temperatures) would increase the background noise/vibration in the anechoic chamber. Hence we did not use laser vibrometers for the bioassays in this study.

Supplementary Information B Background information on discrete wavelet transform (DWT)

The DWT, is defined as

$$X[n, a^j] = \sum_{i=0}^{N-1} x[i]\phi_j^*[i - n] \tag{1}$$

where $x[n]$ $(n = 0, \ldots, N-1)$ is the ant signal, $s = a^j$ is the scale, $a = 2$ for dyadic decomposition, j is the discrete level decomposition and $\phi_j[n]$ is the discrete wavelet

$$\phi_j^*[n] = \frac{1}{\sqrt{a^j}}\phi\Big(\frac{n}{a^j}\Big) \tag{2}$$

which consists of a scaled version of the mother wavelet $\phi(t)$, which can be selected to match with the expected signal. The discrete wavelet transform DWT is an analysis - synthesis technique for which j different time signals $X[n, a_j]$ of mixed even non-stationary signals with different scale information can be separated (e.g. impulses). Most DWT wavelets can be computed in a very efficient way using digital filtering techniques (filter banks). Once the undesired part of the signal is identified, wavelet scales can be recombined, when proper decimation, filtering and interpolation processes are applied to obtain a filtered approximation of $x[n]$ (synthesis phase).

Supplementary Information C Parametric modelling of the wood response

Please note that in a circular membrane like those pieces of wood used, resonance frequencies are not harmonic and depend on the zeros of the first order Bessel functions, which exhibits a near periodicity only for higher modes. Damping of higher modes is very large and the signal is most likely to be obscured by noise and its detection is very difficult. Damping due to humidity of absorption of the wood was minimised by keeping the relative humidity of the air in the anechoic chamber to less than 28% on average. A good approximation of the signal was obtained using a 5th order model (two real sinusoids), so a linear filter has been defined for the substrate. The main advantage of parametric modelling over stationary models (Fourier techniques or lower-order statistical models) is that an accurate model of the signal can be obtained already by using a only a small number of samples (i.e. signals such as non-stationary signals e.g. impacts). As a consequence a particular model can be obtained for each substrate sample using an excitation signal produced by the ants and this particular model can be applied to the same scenario to reduce signal distortion and increase ant signal detection sensitivity, whereas the particular substrate remains unchanged. Resonances of the wood may be modelled as a linear system that produces a set of exponential damped sinusoids (as given in Equation (3)), contaminated with noise

from the acquisition system. Two sinusoids are employed here which cover two vibration modes.

$$x[n] = \sum_{i=1}^{M} a_i \exp(s_i n) + w[n] \quad (3)$$

$x[n]$ is the modelled signal, $w[n]$ is noise (white: GAUSSIAN, zero mean; and wide-sense stationary [3]), M is the model order or number of complex exponentials, $a_i = |a_i|e^{j\phi_i}$ are complex amplitudes (real amplitudes $|a_i|$ & phase shifts ϕ_i), and $s_i = \alpha_i + j2\pi f_i$ are complex parameters (α_i exponentially damped sinusoidal damping coefficients, f_i normalised frequencies with respect to sampling frequency, i.e. normalised HERTZ).

However, the signals recorded with an accelerometer in the anechoic chamber are non-stationary and may be contaminated by distortion or noise. Distortion is due to two sources: a low frequency component owing to variations of the reference level of the accelerometer or ant motion and higher frequency oscillations of the substrate. Continuous wavelet decomposition enables all relevant signal information to be identified in the range of scales $2 - 22$, so that removal of higher scales increases the signal-to-noise ratio and removal of lower scales reduces the low frequency distortions. Compared to other spectral techniques, wavelet processing techniques allows a better treatment of signals with time discontinuities and fast changing phenomena, such as impulses. Substrate resonances produces oscillations in the recorded signals and are difficult to characterise with analytical models because of the variability of the material properties of wood [4]. These oscillations require a broad range of frequencies or scales which overlap with ant signal range, so spectral or wavelet filtering would damage the original signal. It is possible to separate the excitation of the substrate response if enough of the model (motion) is known (e.g. by correlating the action that is measured vibration with a recorded video) and the substrate response (resonances) is known. Here, resonances have been modelled as linear systems which produce noisy sets of exponential damped sinusoids.

Analysis of ant vibration signals

Figure S3. Analysis of signals. Synthesised response of the model (filtered response, Figure 2 main document) and its de-convoluted signal (extracted excitation) for **A** the scratching sound only (Figure 5**B**, main document) and **B** the carrying and dropping of a stone (Figure 5**C**, main document)

The excitation signals depicted in Figure S3**A** and **B** of the scratching/biting response (Figure 5**B**,

main document) and the stone carrying/dropping response (Figure 5**C**, main document) show that the influence (distortion) of the veneer disc's response has been attenuated at the same time the noise is reduced, producing clean excitation signals, previously obscured owing to their weak nature. However, the excitation is much more complex and the ant behaviour is not as clear as with the impact model. Rather than scratching it is likely that the ant is biting and pulling; while for the stone being dragged over the veneer a higher order model with broadband characteristic seems to be necessary as friction is likely to be involved [5].

References

1. Bies DA, Hansen CH (2009) Engineering Noise Control: Theory and Practice. Spon Press, Abingdon.

2. Fletcher NH, Rossing TD (1998) The physics of musical instruments. Springer-Verlag New York.

3. Priestley MB (1988) Non-linear and Non-stationary Time Series Analysis. Academic Press, London.

4. Oberst S, Lai JCS, Evans TA (2013) Novel method for pairing wood samples for choice tests. PLoS ONE (accepted for publication, doi 10.1371/journal.pone.0088835).

5. Akay A (2002) Acoustics of friction. Journal of the Acoustical Society of America 111: 1525-1548.

List of Figures

S1 **Experimental setup to measure the mobility of the veneer disc in the ant-box resting on foam.** Scanning laser vibrometer (PSV-400); loudspeaker; and ant-box minus rectangular container, Figure 1, main document. 1

S2 **Characterisation of veneer discs over mobilities.** Mobilities measured for systems **1**, **2** and **3**; X_i and Y_i stand for the veneer discs' later use as control and treatment sides of system $i = 1, 2, 3$, respectively 1

S3 **Analysis of signals.** Synthesised response of the model and its de-convoluted signal for **A** the scratching sound only and **B** the carrying and dropping of a stone 4
